# Editorial: Science to Foster the WHO Air Quality Guideline Values

**DOI:** 10.3389/ijph.2024.1608249

**Published:** 2025-01-06

**Authors:** Hanna Boogaard, Pallavi Pant, Nino Künzli

**Affiliations:** ^1^ Health Effects Institute, Boston, MA, United States; ^2^ Department of Epidemiology and Public Health, Swiss Tropical and Public Health Institute, Basel, Switzerland; ^3^ University of Basel, Basel, Switzerland; ^4^ Swiss School of Public Health (SSPH+), Zürich, Switzerland

**Keywords:** air pollution, health, WHO air quality guidelines (AQG), policy, legislation

Air pollution is among the leading risk factors for poor health worldwide – in 2021, it was the second leading risk factor for premature mortality, surpassed only by high blood pressure, and resulting in 8.1 million deaths [1]. Everyone is vulnerable to its impacts, and some are more at risk than others. People’s level of vulnerability is outside of individual control, as it evolves with age, health condition, socio-economic status, as well as where people live, study, or work. The impacts of poor air quality can be further exacerbated through exposure to a variety of climate hazards. Rising temperatures are worsening air pollution and its health effects, underscoring the urgent need for integrated action to simultaneously improve air quality and reduce greenhouse gas emissions [2]. Just in the last few years, wildfires, extreme heatwaves, and more frequent and severe dust storms have proven to be devastating to air quality in regions around the globe.

There is a large global body of evidence linking exposure to air pollution, especially fine particulate matter (PM_2.5_), with impacts on all major human organ systems. Furthermore, epidemiological studies have now documented health effects at levels below current national ambient air quality standards. The Health Effects Institute recently completed a comprehensive research initiative to investigate the health effects of long-term exposure to low levels of air pollution in Europe, Canada and the United States [3]. Particular strengths of the studies included the large populations (7–69 million people), state-of-the-art exposure assessment methods, and thorough statistical analyses that applied novel methods. All three studies documented positive associations between mortality and exposure to PM_2.5_ at levels as low as 4 μg/m^3^ or even lower. Furthermore, the studies observed linear (United States), or supra-linear (Canada and Europe) exposure-response functions for PM_2.5_ and mortality, with no evidence for a threshold. This research initiative provided important new evidence of the adverse effects of long-term exposures to low levels of air pollution at and below current standards, suggesting that further reductions in air pollution could yield larger benefits than previously anticipated [3].

Based on these and other studies, the World Health Organization (WHO) released new Air Quality Guidelines (AQG) in September 2021. They recommended that annual mean concentrations of PM_2.5_ should not exceed 5 μg/m^3^, finding that adverse health effects occur above this concentration [4]. They also recommended a set of interim targets, meant to provide a step wise pathway towards achievement of the AQG values set at 35, 25, 15, and 10 μg/m^3^. Governments in the United States and Europe have recently moved toward more stringent PM_2.5_ standards—9 and 10 μg/m^3^, respectively—to align more closely with the 2021 WHO AQG [5, 6]. Meanwhile, the Federal Commission of Air Hygiene advised the Swiss Government to adopt the new WHO AQG values as the national standards [7]. Others such as Uganda have recently adopted National Air Quality Standards for the first time [8], and Brazil has adopted the National Air Quality Policy with progressive air quality targets consistent with the 2021 WHO AQG [9].

**FIGURE 1:**
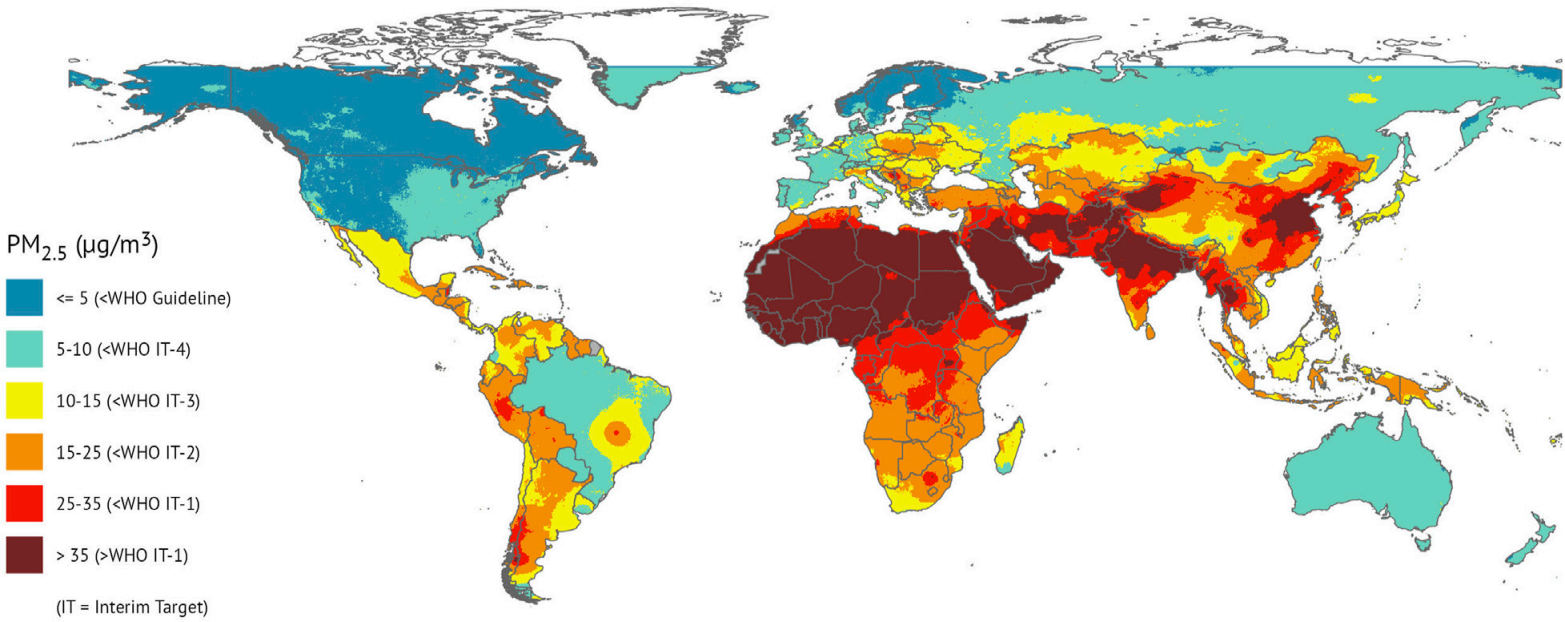
National population-weighted annual average PM_2.5_ concentrations in 2020 [1].

This Special Issue, entitled “*Science to Foster the WHO Air Quality Guideline Values*,” presents recent science that underpins the WHO AQG and offers insights into pathways for action. One issue is abundantly clear—the disease burden from air pollution is not borne equally across the world, with countries in Asia, Africa, and the Middle East experiencing the highest levels of ambient PM_2.5_ and associated health impacts (Figure 1). Hence, there is a particular need to improve air quality in those regions (e.g., Safi et al. and Kundu et al.).

Much of what is currently known about the adverse effects of ambient air pollution and its solutions come from studies conducted in high-income regions, especially North America and Western Europe, with relatively low air pollution levels, and in more recent years, from studies in China where air pollution levels are relatively high [10, 11]. As governments around the world act to improve air quality, there is a continuing need for research to strengthen the local evidence base on disease risk at relatively high levels of air pollution, identify the air pollution sources most responsible for disease burden and assess the public health effectiveness of actions taken to improve air quality. Such studies are also invaluable for strengthening local scientific and infrastructure capacities, raising awareness of local communities, and supporting evidence-based decision making. To strengthen awareness, there is also a need to update Air Quality Index tools – used by many authorities to communicate the state of air quality on a daily basis – with the 2021 WHO AQG (Adebayo-Ojo et al.). More research is also needed to capture the direct and indirect health effects of climate change more fully, including the interactions with air pollution.

Overall, bold air quality and climate actions are needed at all levels–international, national, local–and across all sectors such as transport, energy, industry, agriculture, and residential. There is cause for optimism: there are various examples from locations across the globe that show that if action is taken to improve air quality, so does population health [12]. Scientific data and evidence such as that presented in the articles in the Special Issue, will continue to play a fundamental role in fostering evidence-based air quality and climate actions, to reduce the inequity in air quality both within and across countries, and to close the gap between national air quality standards and the 2021 WHO AQG.
